# Trust toward humans and trust toward artificial intelligence are not associated: Initial insights from self-report and neurostructural brain imaging

**DOI:** 10.1017/pen.2022.5

**Published:** 2023-03-21

**Authors:** Christian Montag, Benjamin Klugah-Brown, Xinqi Zhou, Jennifer Wernicke, Congcong Liu, Juan Kou, Yuanshu Chen, Brian W. Haas, Benjamin Becker

**Affiliations:** 1 Department of Molecular Psychology, Institute of Psychology and Education, Ulm University, Ulm, Germany; 2 Clinical Hospital of Chengdu Brain Science Institute, MOE Key Laboratory for Neuroinformation, School of Life Science and Technology, University of Electronic Science and Technology, Chengdu, China; 3 Institute of Brain and Psychological Sciences, Sichuan Normal University, Chengdu, China; 4 Department of Psychology, Xinxiang Medical University, Henan, China; 5 Department of Psychology, University of Georgia, Athens, GA, USA

**Keywords:** Artificial intelligence, Trust, MRI

## Abstract

The present study examines whether self-reported trust in humans and self-reported trust in [(different) products with built-in] artificial intelligence (AI) are associated with one another and with brain structure. We sampled 90 healthy participants who provided self-reported trust in humans and AI and underwent brain structural magnetic resonance imaging assessment. We found that trust in humans, as measured by the trust facet of the personality inventory NEO-PI-R, and trust in AI products, as measured by items assessing attitudes toward AI and by a composite score based on items assessing trust toward products with in-built AI, were not significantly correlated. We also used a concomitant dimensional neuroimaging approach employing a data-driven s*ource-based morphometry* (*SBM*) analysis of gray-matter-density to investigate neurostructural associations with each trust domain. We found that trust in humans was negatively (and significantly) correlated with an SBM component encompassing striato-thalamic and prefrontal regions. We did not observe significant brain structural association with trust in AI. The present findings provide evidence that trust in humans and trust in AI seem to be dissociable constructs. While the personal disposition to trust in humans might be “hardwired” to the brain’s neurostructural architecture (at least from an individual differences perspective), a corresponding significant link for the disposition to trust AI was not observed. These findings represent an initial step toward elucidating how different forms of trust might be processed on the behavioral and brain level.

Artificial intelligence (AI) represents a key technology, which is in-built in a growing number of products people use daily (Lee, 2019). Examples of such products that are critically based on AI technology are Apple’s Siri or Amazon’s Alexa. There are also many other products, which will likely be on the mass market in the next few years such as self-driving cars and social robots such as Pepper that are in need of consideration. At present, numerous definitions for AI exist in the field (Monett & Lewis, 2018) including computers “mimicking human mental faculties” (Hopgood, 2005, p. 3), but see also further discussions (Wang, 2019; see comment by Bach, 2020). This study was designed to elucidate how trust in AI may be differentiated from interpersonal trust (i.e., trust in other people), on the behavioral and brain level.

Many scientists predict that AI will impact the lives of people around the globe, and the consequences on the societal level represent a highly debated topic. AI may result in flourishing economies; however, it may also endanger hundred thousands of jobs (Kile, 2013; Makridakis, 2017). Given the promises and perils which will arise from the “AI revolution” (Harari, 2017), it is important to understand the way humans may relate to AI and how these types of attitudes are formed. In this young but relevant research field, Sindermann et al. (2021) published a psychometric measure assessing acceptance and fear of AI (attitude toward artificial intelligence [ATAI]) in English, German, and Chinese language. An initial study employing this measure revealed that the ATAI measure is robustly associated with trusting and using diverse products with in-built AI. Hence, this measure shows external validity and thus might provide a robust measure to quantify trust in AI within socio-psychological and neuroscientific contexts. Sindermann et al. also showed that a Chinese sample displayed a higher acceptance of AI as compared to German and UK samples (perhaps due, in part, to cultural differences in communication and planning of AI strategies in China compared to other countries (Demchak, 2019); for differences and similarities see the work by Bareis & Katzenbach (2021)). The finding of the Chinese sample showing higher acceptance scores for AI than the German sample was also recently replicated (Sindermann et al. 2022), but more research in this area is needed, because within country differences likely exist. This study also demonstrated that fear in AI associated with higher levels of neuroticism suggesting a potential association with behavioral and emotional tendencies in interpersonal contexts.

Elucidating trust in AI is an important step toward better understanding how individuals and societies may adapt to the growing interaction with AI in their lives. Accordingly, a highly relevant item with respect to the acceptance of AI in the ATAI assesses trust in AI (making the ATAI scale relevant for the present study). Trust in humans and in society has received increasing attention during the last years (Evans & Krueger, 2009; Weiss et al., 2021), and trust was particularly relevant with respect to good governance during the COVID pandemic (Devine, Gaskell, Jennings & Stoker, 2021; Kim & Liu, 2022). Beyond that, there are further economic reasons for this interest in trust, as higher trust in society tends to be associated with economic growth (Dincer & Uslaner, 2010; Zak & Knack, 2001).

Several different types of research strategies have been used to elucidate the nature of interpersonal trust. For example, twin studies have shed light on heritability estimates (Cesarini et al. 2008), which showed that individual differences in trust have genetic and environmental components. Hence, although the environment has a substantial share in explaining individual differences in trust, (molecular-)genetic components are also of relevance (Krueger et al., 2012). Behavioral studies have related individual differences in trust behavior to personality traits (i.e., higher extraversion and lower neuroticism; Evans & Revelle, 2008). Personality traits however are both shaped by genes and the environment (Montag, Ebstein, Jawinski & Markett, 2020). Beyond this, endocrinological studies have demonstrated links between oxytocin and trust (Kosfeld, Heinrichs, Zak, Fischbacher & Fehr, 2005; Quintana et al., 2021; Xu, Becker & Kendrick, 2019), however see also the work by Declerck, Boone, Pauwels, Vogt and Fehr (2020). Recently, several neuroimaging methods have been used to examine how the function and structure of the brain correlate with behavioral measures of trust. These studies show that individual differences in trust are linked to variations in the structural architecture of the brain (Aimone, Houser & Weber, 2014; Haas, Ishak, Anderson & Filkowski 2015), whereas twin studies show that the structural and functional architecture of the brain is shaped by both genetics and the environment (Liu et al., 2021; Jansen, Mous, White, Posthuma & Polderman, 2015; Peper, Brouwer, Boomsma, Kahn & Hulshoff Pol, 2007). For example, Haas et al. (2015) showed that higher self-reported trust is associated with increased volume in ventromedial prefrontal cortex areas and the insula. In sum, existing empirical research shows that trust has a biological basis and has been studied in a variety of ways. However, the existing body of trust research is currently limited to the concept of trust as related to other people (i.e., interpersonal trust toward other humans) and has not been investigated as a construct as related to AI or machines. As AI products rapidly permeate in our everyday life, we consider it as an appropriate time to better understand associations between trust in humans and trust in AI on both the behavioral and neurobiological level, which has not been systematically examined so far. From our perspective, the investigation of trusting humans and AI in a single design represents a timely and highly relevant research endeavor. This approach provides the opportunity to examine associations between both domains on the behavioral and brain structural level.

In detail, we ask if humans with higher tendencies toward trusting other humans also exhibit higher levels of trust in machines with in-built AI. This may imply that the same biological pathways may underlie trusting behavior across different contexts. This may not necessarily be the case, because the so-called concept of the “uncanny valley” proposes a non-linear function when describing the patterns of trust in machines that are human like (for a critical review, see Wang, Lilienfeld & Rochat, 2015). In detail, the concept posits that with increasing human-like characteristics of AI the familiarity, likeability, and trust ratings of a machine can only increase up to a certain level. At some point, a small plateau is reached and the ratings then switch into a negative evaluation. This is the case when the machine is very human like but remains distinguishable from a human (Mathur & Reichling, 2016). Therefore, trust formation to different product groups which are different in terms of human-alikeness might argue against the fact that trusting machines might in every case be associated with the same neural pathways as trusting humans. Given the lack of empirical studies in this field, we decided to assess the general trust toward AI (as part of the ATAI measure) separately from the individual trust toward a range of specific products where AI is in-built. These products range from widely used Siri by Apple to human-like androids. Moreover, given that trust in other humans is associated with variation in brain structure (Haas et al., 2015), but trust toward humans might have distinct neural underpinnings compared to trusting AI, we examined in the present study if trust in humans and trust in AI share an overlapping brain structural basis. To this end, we combined magnetic resonance imaging (MRI) brain structural assessments with source-based morphometry (SBM) as a data-driven multivariate approach to brain structural analyses as this demonstrates advantages over the conventional univariate analysis techniques such as voxel-based morphometry (VBM), including higher sensitivity (Gupta, Turner & Calhoun, 2019; Zhou et al., 2022).

## 1. Methods

### 1.1. Participants

Ninety male participants (age = 22.82, SD = 2.25) without a current or a history of a diagnosed psychiatric or neurological disorder were enrolled in the present study. Thus, participants reaching cutoff values for disorders such as depression were not included in the analysis. The sample was part of a neuroimaging project examining associations between individual variations in problematic internet gaming and brain structure (see previous publication by Zhou et al. (2020) using the same sample; see also the replication approach in Klugah-Brown et al., 2022). Within the previous study, we used a dimensional approach to investigate Gaming Disorder and did not investigate patients with a diagnosed Gaming Disorder vs. healthy controls. Participants underwent MRI (T1-weighted imaging) to assess brain structure. Within the context of the present study, participants were asked to complete several questionnaires designed to assess self-reported trust in humans, attitudes toward AI, and trust in several products with in-built AI. The items were presented together with Likert format answers as described below. For further details on the sampling and data acquisition, see the work by Zhou et al. (2020). The study and its procedures had full approval by the local ethics committee at the University of Electronic Science and Technology of China and adhered to the most recent version of the Declaration of Helsinki. All participants provided informed consent.

### 1.2. Attitude toward artificial intelligence

All participants completed the ATAI (Sindermann et al., 2021). The scale consists in total of five items. Two items form the subscale “acceptance of AI” (*α* = .76 in this sample) and three items assess the subscale “fear of AI” (*α* = .65 in this sample). All items are answered via options ranging between 0 = “strongly disagree” and 10 = “strongly agree.” Higher scores correspond to higher acceptance or higher fear of AI. Note that for the present analysis focusing on trust, item 2 of the ATAI (“I trust artificial intelligence”) is of particular interest. Item 2 belongs to the acceptance scale of the ATAI.

### 1.3. Trusting diverse products where AI is in-built (TDP-AI)

All participants completed items assessing trust toward diverse products where AI is in-built (TDP-AI; Sindermann et al., 2021). Products assessed were Google’s self-driving car, Apple’s Siri, the Chinese Alexa (Amazon), the social robot Pepper, and four human-like android products (Erica, Geminoid HI-1, Sophia, Geminoid DK). For the first four products, participants were asked if they would be willing to use them (subscale “willingness to use”) and if they are currently using them (yes/no) but also how much they trust these products. Hence, each product was investigated with three items. For the four human-like androids, it was asked if one would accept them as companions (subscale “interact”) and how much one would trust these androids. Here, each product was investigated with two items.

The trust items as well as the willingness to use/interact items were administered on a scale ranging from 0 = “strongly disagree” to 10 = “strongly agree.” Higher scores indicate more trust toward the product or more willingness to use/interact with the product. Internal consistencies for all items assessing trust toward the respective AI product were (alpha) .93. In the following investigations, we mainly focused on a composite score of all items assessing trust toward product with in-built AI, but also see more fine-grained analysis in the supplementary material.

### 1.4. Trust facet of the personality dimension agreeableness from the NEO-PI-R

Participants also completed the trust (A1) facet of agreeableness within the NEO-PI-R (Costa & McCrae, 2008) in line with how Haas et al. (2015) assessed interpersonal trust in their MRI work. The trust facet is comprised of eight items that are answered on a five-point Likert scale ranging from 0 = “strongly disagree” to 4 = “strongly agree.” A sum score was created (*α* = .71) with higher scores representing higher interpersonal trust. The Chinese version of the trust facet was conducted via forth- and back-translation by two bilingual speaking scientists from our work groups.

### 1.5. Statistical analysis of self-reported trust data

Associations between the self-reported trust data of the NEO-PI-R trust facet, the ATAI, and the TDP-AI were examined by means of correlational analysis. Note that we used a five-point Likert scale to assess individual differences in trust and an eleven point Likert scale in the context of the ATAI and trust in AI product variables. The different scaling use might result in different fine-granular variance levels. Therefore, future studies should also investigate these research questions using the same Likert scales.

**Figure 1.**
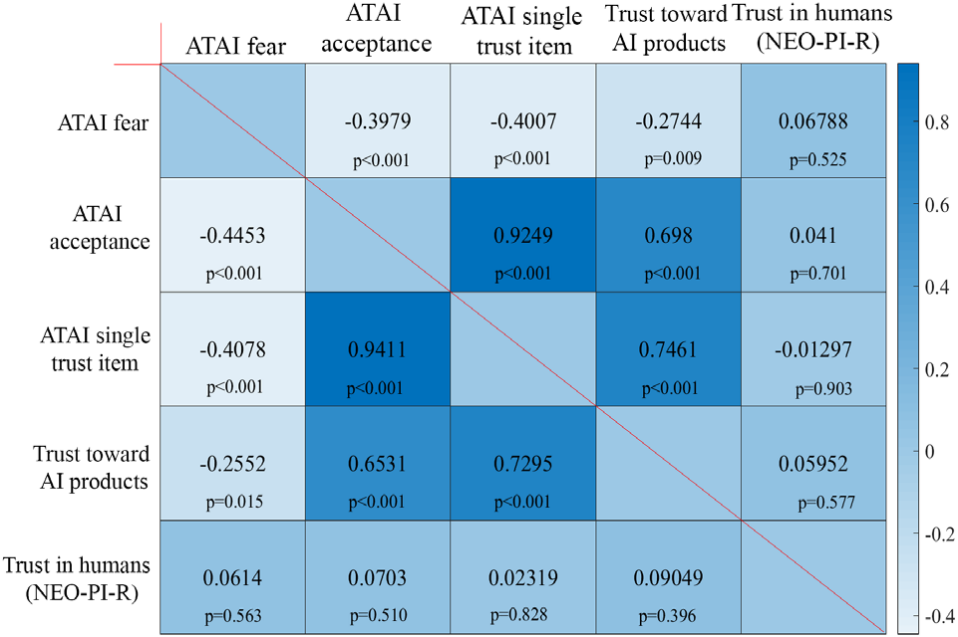
Correlation patterns between the relevant ATAI/trust variables. Upper right half depicts Pearson correlations. Lower left half depicts Spearman correlations. Results indicate that trust in humans (NEO-PI-R) is not associated with trust toward AI or trust in products with in-built AI. Significance is presented on a two-tailed test level.

It was not controlled for age, as age was not significantly associated with the self-report variables NEO-PI-R, ATAI, or composite score of the TDP-AI. Parametric correlation analysis (Pearson) was performed (although the data mostly resembled normal distributions after visual inspections, for reasons of transparency in Figure 1 also Spearman correlations are presented for comparison showing no meaningful differences in terms of differing effect sizes, which were not further tested though). One could question to run linear correlational analysis against the background of the uncanny valley theory. As becomes apparent in Table 1, our data do not support the uncanny valley and we also ran brain–trust correlations on item level. Finally, Bayesian factor correlation analyses were computed to determine the robustness of non-significant associations.

**Table 1.** Descriptive statistics of the self-reported trust/ATAI variables

Trust variables	Mean	Standard deviation	Actual range	History of usage (for four products only)
Trust toward self-driving car	5.94	2.41	0–10	No = 88 (97.8%)
Trust toward Siri	5.87	2.58	0–10	No = 47 (52.2%)
Trust toward Chinese Alexa	5.64	2.34	0–10	No = 77 (85.6%)
Trust toward social robot Pepper	5.67	2.21	0–10	No = 83 (92.2%)
Trust toward Erica	5.83	2.26	0–10	
Trust toward Geminoid HI-1	5.40	2.41	0–10	
Trust toward Sophia	5.47	2.40	0–10	
Trust toward Geminoid DK	5.63	2.47	0–10	
Trust toward all AI products (sum score)	45.46	15.70	3–79	
“I trust AI” (from the ATAI; ATAI Trust in AI)	6.08	2.46	0–10	
ATAI fear	12.23	5.99	0–30	
ATAI acceptance	13.52	3.97	2–20	
Trust toward humans (NEO-PI-R)	19.90	3.98	6–29	

### 1.6. Acquisition of brain structure (T1-weigthed MRI acquisition)

Brain structural data were acquired on a 3.0 T GE MR750 system (General Electric Medical Systems, Milwaukee, WI, USA). T1-weighted high-resolution anatomical images were acquired with a spoiled gradient echo pulse sequence, repetition time (TR) = 6 ms, echo time (TE) = 2 ms, flip angle = 9 degree, field of view (FOV) = 256 × 256 mm, acquisition matrix = 256 × 256, thickness = 1 mm, and number of slices = 156.

### 1.7. MRI data preprocessing

Structural MRI data were preprocessed with CAT12 implementing a computational anatomy approach (http://dbm.neuro.uni-jena.de/cat). Data processing involved the following steps: firstly, T1-weighted images were bias-corrected, segmented into gray matter (GM), white matter (WM), and cerebrospinal fluid, and spatially normalized to the standard Montreal Neurological Institute space. Secondly, GM images were smoothed with a Gaussian kernel of 8 mm full-width at half maximum for subsequent statistical analysis, and the total intracranial volume was estimated to correct for individual differences in brain size.

### 1.8. SBM and statistical analysis regarding MRI data

Gray matter density (GMD) was analyzed by employing SBM as implemented in the GIFT toolbox (http://mialab.mrn.org/software/gift/) (Xu, Groth, Pearlson, Schretlen & Calhoun, 2009). SBM uses independent component analysis (ICA) to extract features reflecting multivariate relationships among GMD regions. Using the minimum description length, the number of components was estimated, and then through ICASSO and further visual inspection, intrinsic components (ICs) were selected to ensure the removal of artifactual components mostly those exhibiting high values in ventricles, WM, and or showing less stability across runs. Associations between brain structural variations and variations in interpersonal trust (human level) and trust in AI were examined by means of multiple linear regression models including the “loading coefficients” of the selected ICs as dependent variable, and ATAI, TDP-AI, and trust toward humans (NEO-PI-R) as independent variables for each model. For reasons of simplicity we focus in the result section in particular on the association between trust in humans and component 1 (and do so with presenting a correlation coefficient). Age did not have a relevant effect on this association and therefore was not controlled for. Significance tests were thresholded at *p* < .05.

## 2. Results

The descriptive statistics indicate that on average participants showed “neutral to slight positive” level of trust toward products with AI (see Table 1).

### 2.1. Associations between trust in AI and trust in humans–correlation patterns

The pattern of correlation among variables indicates that trust ratings toward the diverse AI products (here a combined sum score) were highly intercorrelated (see supplementary material) and the composite score “Trust toward AI products” from the TDP-AI also correlated with the single trust item of the ATAI (“I trust AI,” item 2). The overall pattern of correlation regarding ATAI and TDP-AI measures is consistent with the results of Sindermann et al. (2021). Beyond that, we found that trust toward AI and trust toward humans were not significantly associated (nearly null associations), suggesting unrelated domains (note that some android products show some positive association trends in mild correlation areas, namely those androids, which are designed as Asian-looking like androids; these correlations are presented in the Supplementary Figure S1). Note that aside from Caucasian-looking androids Asian-looking androids were presented, because it has been reported that different level of mere exposure to faces from different ethnic groups can influence likeability (Zebrowitz, White & Wieneke, 2008).

Note that age was not associated with any of the variables in Figure 1 and therefore is not presented within the correlation pattern. We also computed Bayesian factor correlation analyses, which confirmed the lack of associations for trust in humans and trust in AI (Supplementary Table S1).

**Figure 2.**
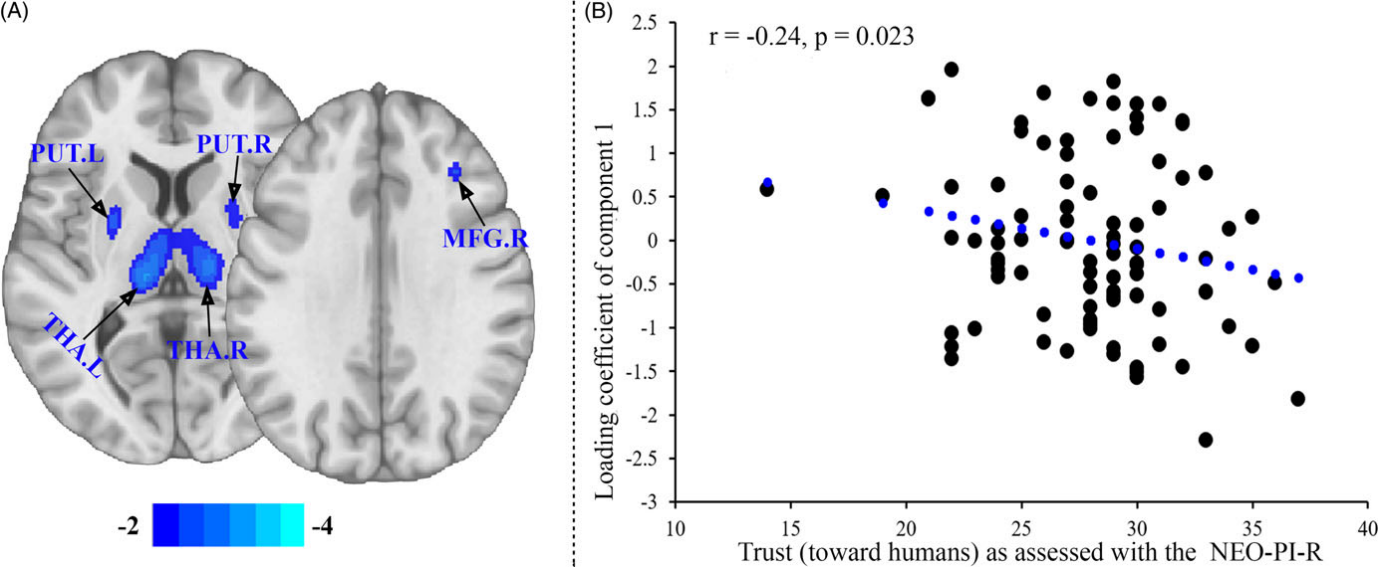
Higher trust in humans is accompanied by lower gray matter density in the bilateral thalamus and dorsal striatum, as well as a right (middle) frontal region of component 1 as shown in (A). The scatter plot (B) shows the association between the significant component and trust in humans. Note that in (B) the Pearson correlation is depicted (of note: Spearman's Rho is −.238, p = .024). R-Square for the regression is rounded 0.06 (hence about 6% explained shared variance).

From the SBM analysis, four ICs were estimated by the ICA. On inspection, all four components estimated from the ICA presented a high quality (i.e., most voxels were located in GM and the ICs presented low spatial overlap). The loading coefficients were extracted from these four ICs and subjected to linear regression analyses which revealed that of the four ICs, only one component (Component 1, as shown in Supplementary Table S3) showed a significant association with trust toward humans. The other ICs were not significantly associated with trust toward humans. Examining associations between the ICs and individual variations in attitude toward AI (ATAI acceptance; ATAI fear), the item “I trust artificial intelligence” (ATAI single trust item) or the composite trust score combining trust across the eight AI products (trust toward AI products) did not yield significant results. Higher trust in humans was negatively associated with lower GMD in the bilateral thalamus and dorsal striatum, as well as a right (middle) frontal region of component 1 (Figure 2A; 2B). The component map in Figure 2A was obtained on the whole brain level, and we initially applied a *p*-value computation on the voxel level with a subsequent application of an FDR = 0.05 approach for multiple comparisons correction at the voxel level. In addition, given that our previous study encompassed only *n* = 82 subjects because of stricter inclusion criteria (Zhou et al., 2020) we considered it mandatory to repeat the present analysis with the identical sample. To this end, we recalculated the ICA with the 82 subjects and compared their loading coefficient values. We found no significant differences in the values or in the spatial components (Supplementary Figure 2, Table S2).

## 3. Discussion

The present work examined the relationship between trust toward humans and the general trust toward AI as well as trust in products with in-built AI on a behavioral and brain structural level. In line with Sindermann et al. (2021), general trust in AI as assessed by the ATAI scale is robustly linked to trusting products with in-built AI, indicating a high external validity of the construct. Although previous observations suggest that the ATAI scales are associated with individual variations in behavioral tendencies such as neuroticism (Sindermann et al., 2022), the present study did not observe a relationship between trust in humans and trust in AI in general or trust in AI products, respectively. Further examination of structural brain data revealed that variations in trust in AI were not significantly associated with individual variations in brain structure. But we observed that higher trust in humans was negatively (and significantly) associated with an SBM GMD component spanning bilateral thalamic-striatal regions and the right middle frontal cortex. Summarizing, the present findings suggest that trust in humans and trust in AI are not associated on the self-report level. But variations in interpersonal trust are associated with GMD variations, while no corresponding significant observation was made for trust in AI.

The lack of significant associations between the scales assessing individual variations in trust in AI and trust in humans provides initial tentative support that these trust domains are not related with each other. Whereas trust in other humans facilitates cooperation and exchange in social groups and may represent an evolutionary evolved survival advantage, exposure to and experiences with AI represent a very recent phenomenon. We hypothesized that variations in the two domains might be associated, possibly reflecting a common trust factor. But we did not observe such an association. This might, however, change in the near future, when new products are developed, which get more and more human like. At least we observed a small, but not significant trend that higher trust toward humans is associated with higher trust toward androids belonging to the same ethnicity of the study participants, hence human like appearing products of the same ethnic group. However, a corresponding dip in trust toward human-like androids (hence the uncanny valley), which are getting very close to a human being, was not mirrored in the descriptive statistics of the present study and could also not be really investigated as we did not ask for trust toward a specific human person.

The lack of an association between trust in humans and trust in AI products was additionally mirrored on the structural brain level. While higher levels of trust in humans as assessed by the NEO-PI-R trust scale were significantly related to reduced GMD in a component spanning the bilateral thalamus and dorsal striatum as well as some middle prefrontal regions, no significant associations with scales assessing individual variations in trust toward AI or AI products were observed. In line with the present findings, previous studies reported associations between individual variations in self-reported trust toward humans or interpersonal trust behavior and the structural architecture of the brain (Feng et al., 2021; Haas et al., 2015). In particular, Haas et al. (2015) employed the identical human trust measure as in our study and by means of a univariate VBM analysis observed *positive* associations between higher trust in humans and higher GM volume in medial frontal and insular regions. In contrast, the present study observed that *increased* trust was associated with reduced GM *density* of the bilateral thalamus and dorsal striatum, while Haas et al. (2015) found increased trust was associated with increased GM *volume* of the ventromedial prefrontal cortex. The difference in results may be accounted for by methodological differences across studies and/or cultural differences across samples. For example, the current study employed a multivariate data-driven SBM approach assessing GMD while the previous study by Haas et al. (2015) employed a conventional univariate VBM analytic approach assessing GM volume. Variations in the analysis and even the preprocessing of structural brain data have increasingly been associated with variations in the identified brain regions and may contribute to replicability issues with respect to associations between personality or behavioral domains with indices of regional brain structure (e.g., Valk et al., 2020; however, see also Becker et al., 2015; Liu et al., 2021; Zhou et al. 2022). Furthermore, in the current study we examined a sample of Chinese participants, while the study by Haas et al. (2015) investigated a sample of American participants. A large body of evidence demonstrates that Chinese and Americans differ in terms of interpersonal relationships (Wei, Carrera, Su, Lin & Yi, 2013) and several aspects of trust (Huang & Rau, 2019; Huff & Kelley, 2003; Klein et al., 2019; Özer, Zheng & Ren, 2014). Taken together, the way people construe trust as a generalized construct or specifically within interpersonal relationships is likely influenced by many factors including the cultural context but also contextual and social factors within a culture. The general tendency to trust may however represent an underlying behavioral tendency which influences trust across contexts. Finally, we mention that the present discussion could also benefit by including findings from neurostructural investigations of empathy, as empathy (in particular empathic concern) is related to trust (Kamas & Preston, 2021). In a work on empathy and the structure of the human brain (Banissy et al., 2012), it has been observed that more empathic concern was associated with lower GM volume of the precuneus and anterior cingulate (region of interest analysis). The whole brain analysis also revealed a negative association between empathic concern and the left inferior frontal gyrus. The latter finding is interesting in the context of our findings where we observed such a negative association between higher trust and lower GM volumes of the *right* (middle) frontal regions. Clearly bringing in together the constructs of empathy, trust toward humans and AI in one single research design will be of interest to better understand similarities and differences in the constructs (also on brain structural level) soon.

How can the present brain-interpersonal-trust associations be explained in terms of their functional relevance for trust? The brain structures being associated in the present work with interpersonal trust are part of established thalamo-striato-cortical loops which have been strongly involved in cognitive control and impulsive behavior (den Heuvel et al., 2010; Robbins, Gillan, Smith, de Wit & Ersche, 2012). Of note, an intriguing psychological theory (Murray et al., 2011) distinguishes between the existence of more impulsive and more deliberate trust, with the present brain structural associations suggesting that the NEO-PI-R trust scale may stronger touch upon the impulsive nature of trust. Although the NEO-PI-R’s trust facet has not been constructed to disentangle more impulsive and more deliberate trust, it might be the case that the more impulsive – hence low cognitive and high automatic – trust behavior might go along with lower brain density within the thalamo-striato-cortical loops whereas more deliberate – hence high self-control – trusting behavior (see also Evans, Dillon, Goldin & Krueger (2011)) might be linked to higher regional density in other circuits supporting deliberate decisions to trust.

In contrast to interpersonal trust, individual variations in AI trust were not significantly associated with variations in brain structure. The absence of significant structural brain correlations with the attitude toward AI scale (including trust in AI) may be explained in terms of learning experience or a hard-wired architecture of the brain. Although speculative, it is for instance conceivable that repeated experience during social interactions – in interactions with our genetic make-up (see the aforementioned twin study (Cesarini et al., 2008) and several discussions about gene by environment effects in the introduction) – forms trust in humans and concomitantly shapes the underlying brain structure. Alternatively, it is conceivable that trust in other humans in terms of an evolutionary adaptive function may be related to individual variation in genetics and brain structure per se. Both (highly speculative) explanations can also account for the lack of significant brain structural associations with trust in AI, such that AI represents a very new phenomenon and corresponding experiences are not sufficient to strongly imprint brain structure. Moreover, acceptance or fear of AI might be diffuse and as an attitude too easily be changed to be robustly (here meaning significantly) linked to brain structure differences.

The present study represents an initial and highly exploratory approach to map associations between trust in humans and trust in AI on the self-report and structural brain level. The findings should be cautiously interpreted in terms of the following limitations of the study. First, the present study was conducted only with male participants; therefore, future studies should investigate whether the results generalize to females. Second, the trust variables were assessed via self-report, which comes with the usual potential problems such as answering in a socially desirable way or lacking insights into one’s own person. Trust can be for instance more objectively assessed with the trust game (Civai & Hawes, 2016). In every case, we would favor objective trust data in future studies. Third, this field is very new, and we do not know how stable attitudes toward AI are. We know that personality traits are rather stable (for a more balanced view beyond this simplification, see the work by Wagner, Orth, Bleidorn, Hopwood & Kandler, 2020), for attitudes toward AI or trusting AI this is not clear, and changes in the ATAI would be also of interest to be investigated in the context of brain data. The study is further limited by only having assessed product usage (if possible), but not the degree of familiarity, which could impact trust levels via mere exposure levels. Furthermore, the effect size representing the association between NEO-PI-R’s trust scale and the brain structural data was small and therefore warrants replication. In particular such a replication is also needed against the background of several constructs being investigated in the present brain structural study. Finally, the present study has been conducted in a Chinese sample. We already know from two former works that acceptance of AI seems to be higher in Chinese samples compared to samples from other countries (i.e., Germany). Therefore, investigating the present research question in samples from other countries and cultures might result in different insights. Moreover, future studies might want to also achieve a larger sample size, although the MRI part makes such an endeavor more difficult. Finally, our findings might be biased by individuals showing different familiarity levels with AI and therefore future studies should also assess such a familiarity with AI variables.

In sum, to our knowledge this work is one of the first of its kind to investigate both trust in humans and trust in AI in one study design also relying on brain imaging. Both trust constructs appear not to be related to each other and while a significant brain-trust association with trust toward humans could be observed, this was not the case for trust toward AI. Against the background of the raised limitations, the present findings should be considered as preliminary. Replication studies demonstrating the replicability of the findings in independent samples and across different brain structural analyses methods (Zhou et al. 2022) are key to establish the robustness of the present findings. We would be happy to see other scientists joining this timely research area, which likely will gain rapidly in relevance in an age where AI is more and more in-built in evermore products. Insights from research endeavors such as the present one help to improve our understanding of interacting with AI, and this may ultimately be relevant knowledge to improve human–machine interaction, being of growing relevance in digital connected societies.

## Supplementary material

For supplementary material accompanying this paper visit https://doi.org/10.1017/pen.2022.5. click here to view supplementary material
