# Supplementary material for: Trust toward humans and trust toward artificial intelligence are not associated: Initial insights from self-report and neurostructural brain imaging
Source: Personal Neurosci. 2023 Mar 21;6:e3. doi: 10.1017/pen.2022.5 (PMC10725778; doi:10.1017/pen.2022.5)
Supplement: Supplementary file 1 [file pensup.zip › S2513988622000050sup002.docx]

**Supplementary Material**

Table S1: Bayesian factor Pearson correlation analysis for fear and trust

| Variables | ATAI fear | ATAI acceptance | ATAI single trust item | Trust towards AI product | Trust in humans (NEO-PI-R) |
| --- | --- | --- | --- | --- | --- |
| ATAI fear | - |  |  |  |  |
| BF_10_ | inf |  |  |  |  |
| p |  |  |  |  |  |
| ATAI acceptance | r=-0.398 | - |  |  |  |
| BF_10_ | 151.349 |  |  |  |  |
| p | <0.001 |  |  |  |  |
| ATAI single trust item | r=-0.401 | r=0.925 | - |  |  |
| BF_10_ | 169.976 | 3.06E+35 |  |  |  |
| p | <0.001 | <0.001 |  |  |  |
| Trust towards Ai product | r=-0.397 | r=0.698 | r=0.746 | - |  |
| BF_10_ | 2.520 | 3.56E+11 | 1.98E+14 |  |  |
| p | =0.009 | <0.001 | <0.001 |  |  |
| Trust in humans (NEO-PI-R) | r=0.068 | r=0.041 | r=-0.013 | r=0.059 | - |
| BF_10_ | 0.102 | 0.090 | 0.084 | 0.097 |  |
| p | =0.525 | =0.701 | =0.903 | =0.577 |  |

BF; Bayesian factor, * BF₁₀ > 10, ** BF₁₀ > 30, *** BF₁₀ > 100


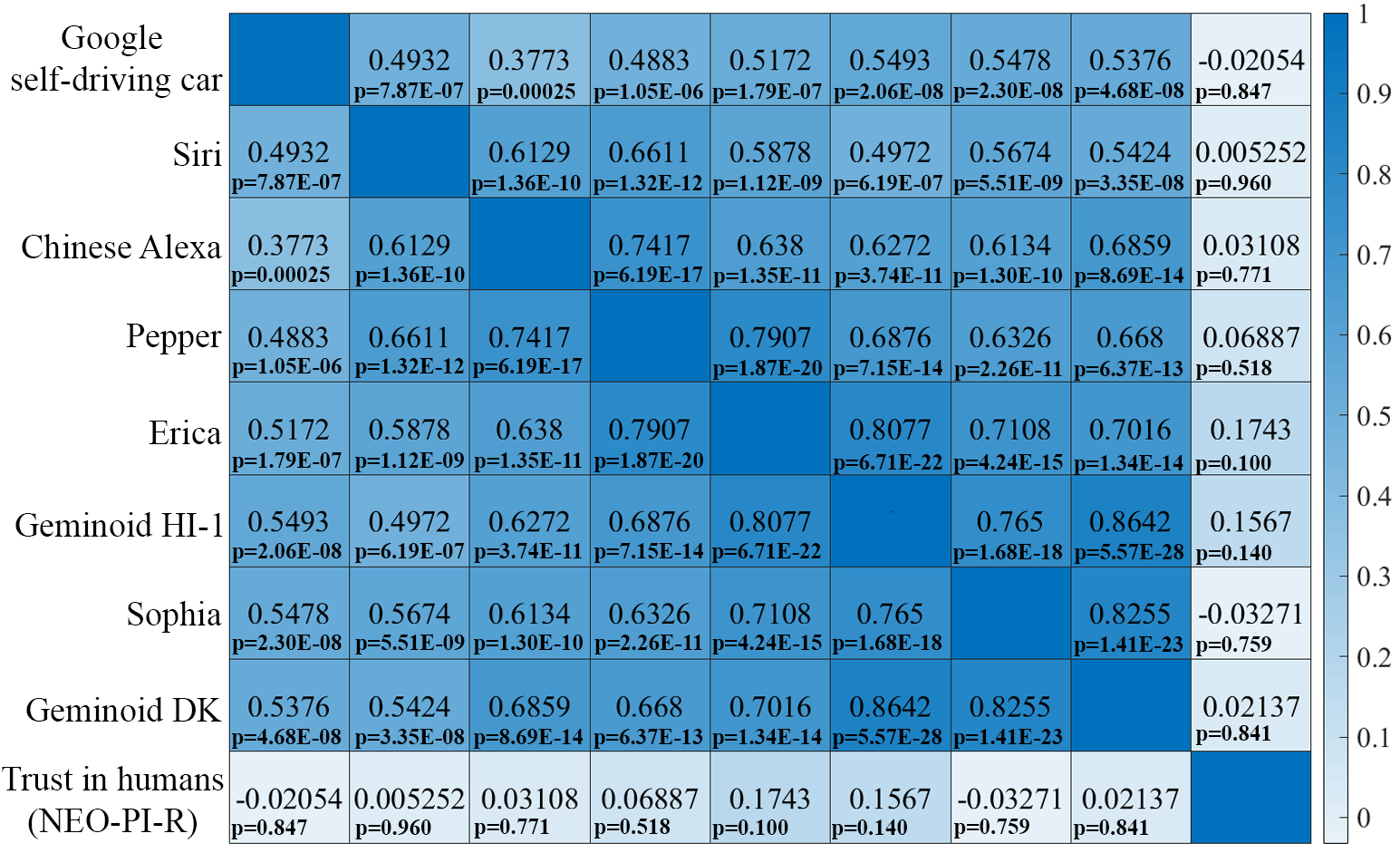


Figure S1: Pearson correlation analysis for trust in eight AI product and humans(two-tailed tested). E; Exponent (10^x^)


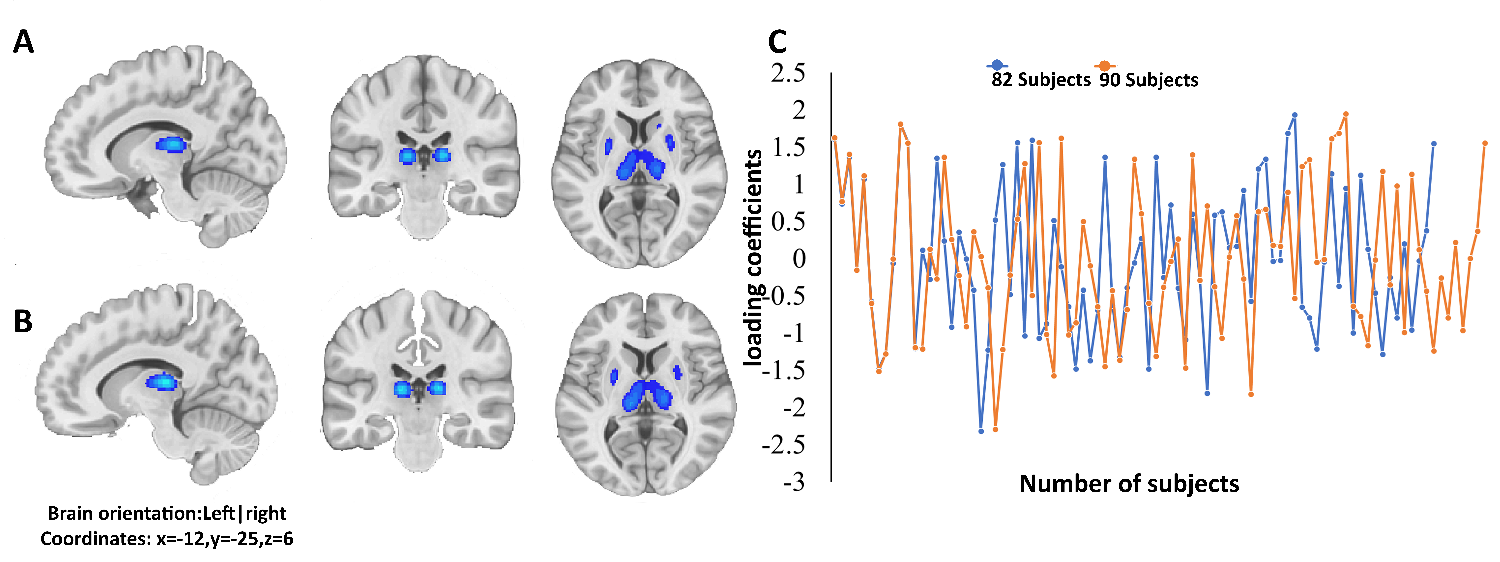


Figure S2: Re-computation of ICA two data sets. A) demonstrates the significant component estimated from 82 subjects (subjects used in the previous work [31]). B) shows the significant component derived from all 90 subjects. C) shows a comparative test of the component loading coefficients from 82 and 90 subjects respectively.

Table S2: Statistical t-test between 82 and 90 subjects using their loading coefficients

| VARIABLES | 82_subjects | 90_subjects |
| --- | --- | --- |
| Mean | -0.018 | 5.680E-09 |
| Variance | 0.982 | 1.00 |
| Observations | 82 | 90 |
| Pooled variance | 0.991 |  |
| Hypothesized mean difference | 0 |  |
| Df | 170 |  |
| T stat | -0.124 |  |
| P(t<=t) one-tail | 0.450 |  |
| T critical one-tail | 1.653 |  |
| P(t<=t) two-tail | 0.901 |  |
| T critical two-tail | 1.974 |  |

Table S3: Table showing all four components as estimated by the ICA-SBM

| Component number | Maps | Results from the regression analysis revealed the following associations with trust in humans (p<0.05) |
| --- | --- | --- |
| 1 | 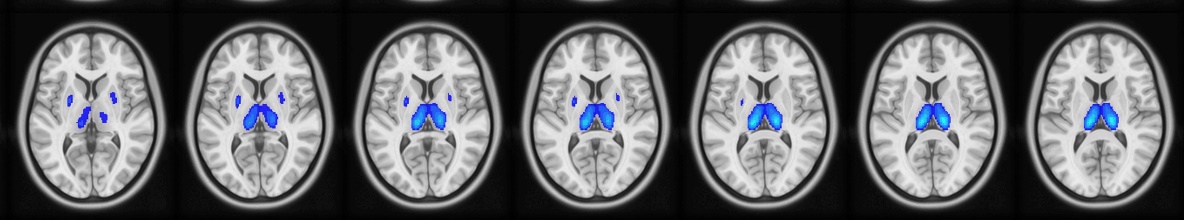 | Significant |
| 2 | 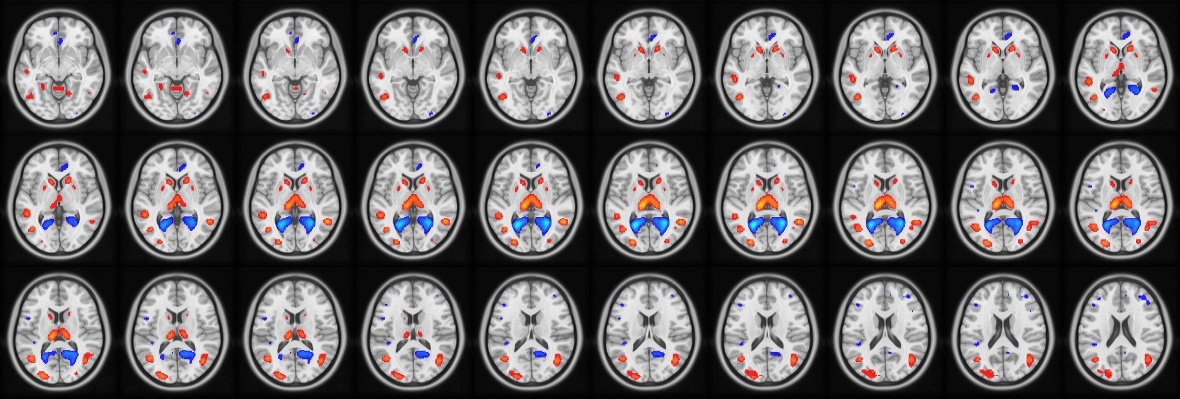 | Not significant |
| 3 | 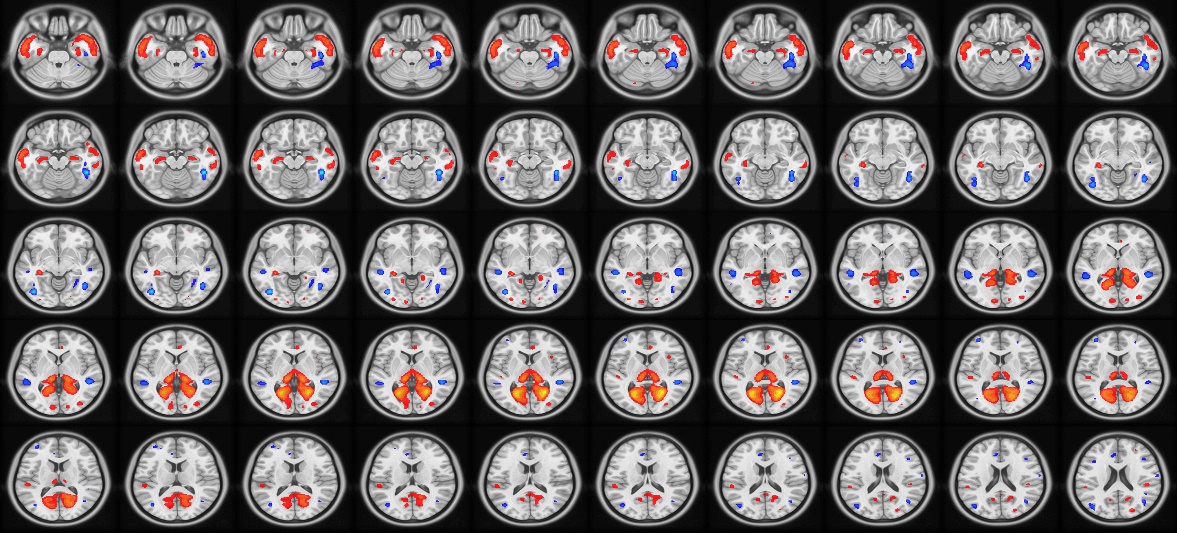 | Not significant |
| 4 | 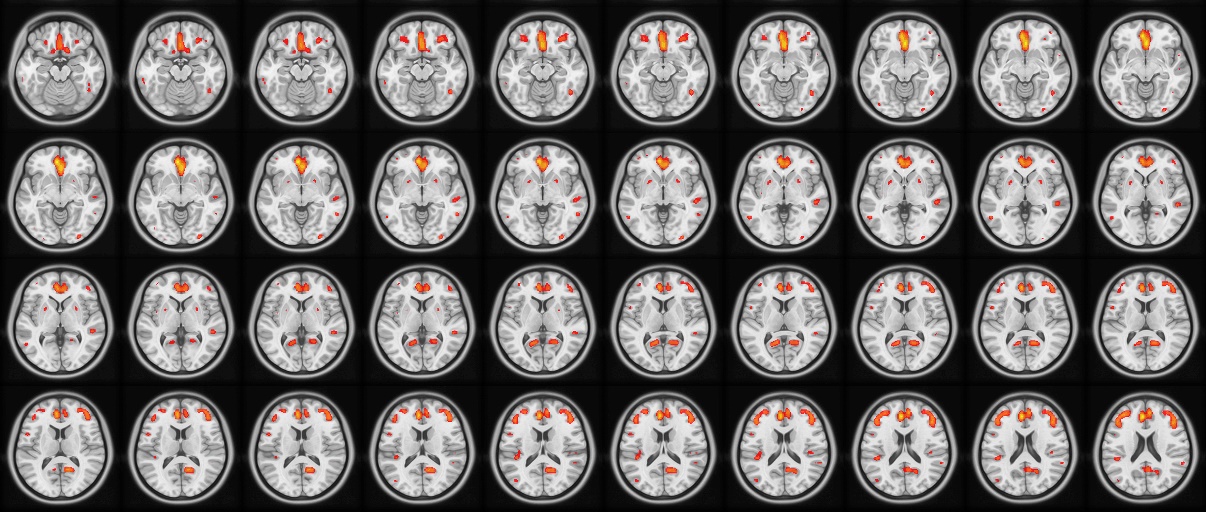 | Not significant |
