# Supplementary figures and images for: Trust toward humans and trust toward artificial intelligence are not associated: Initial insights from self-report and neurostructural brain imaging
Source: Personal Neurosci. 2023 Mar 21;6:e3. doi: 10.1017/pen.2022.5 (PMC10725778; doi:10.1017/pen.2022.5)

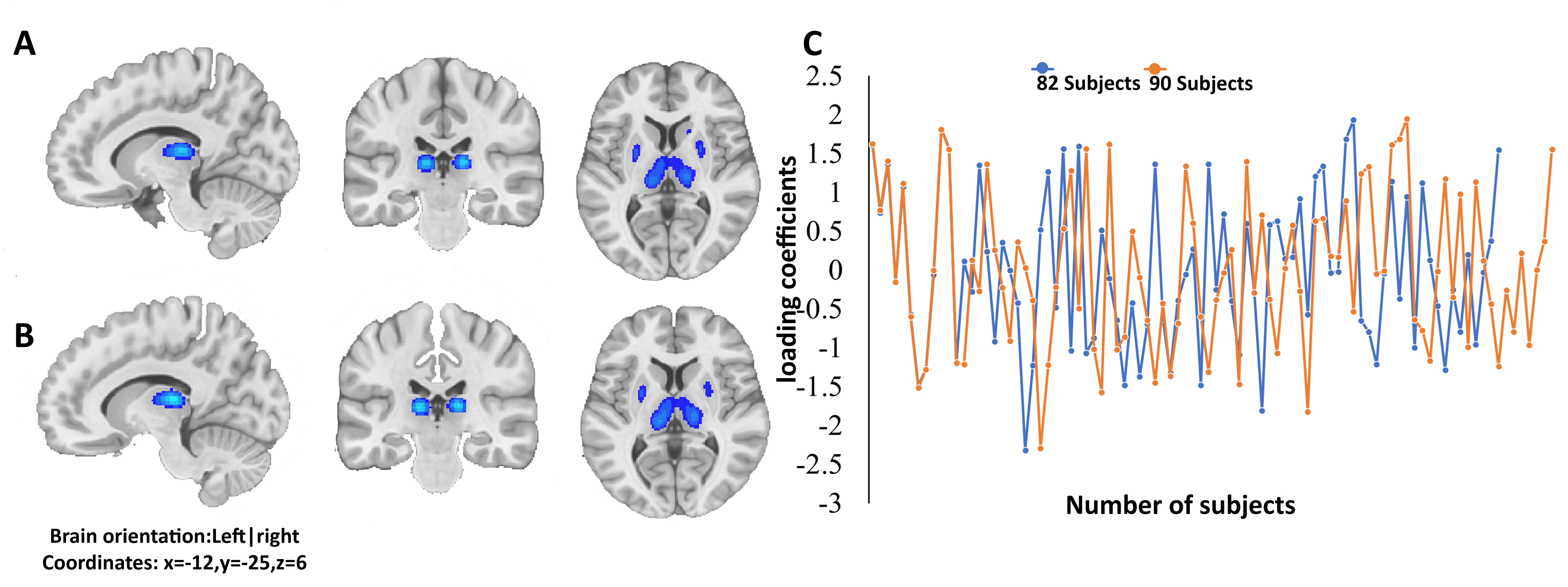

Supplement: Supplementary file 1 [file pensup.zip › S2513988622000050sup001.tif]

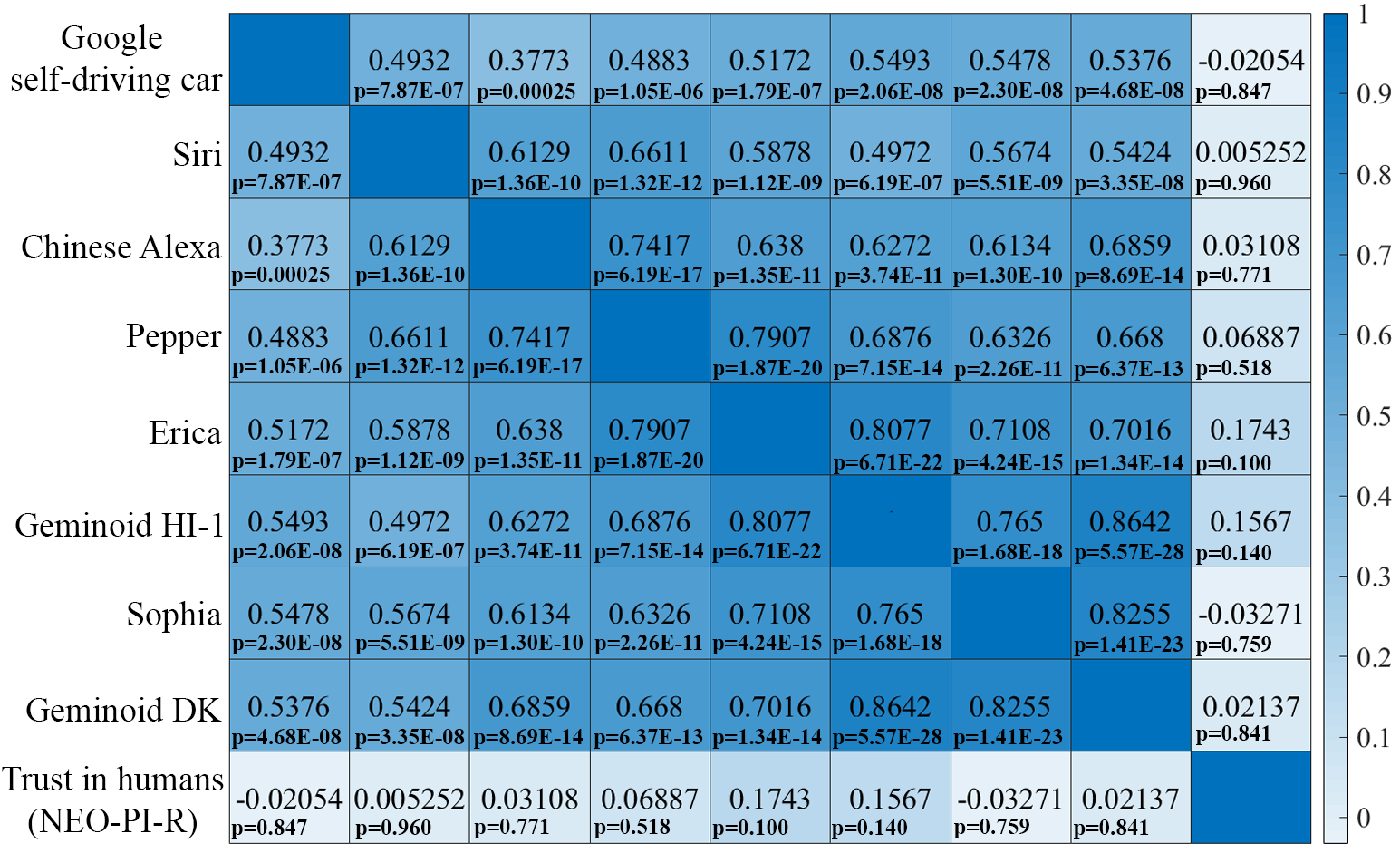

Supplement: Supplementary file 1 [file pensup.zip › S2513988622000050sup003.tif]
